# Cost-effectiveness of the 3E model in diabetes management: a machine learning approach to assess long-term economic impact

**DOI:** 10.3389/fpubh.2025.1571546

**Published:** 2025-05-23

**Authors:** Supriya Raghav, Santosh Kumar, Hamid Ashraf, Poonam Khanna

**Affiliations:** ^1^Department of Public Health, School of Public Health, Poornima University, Jaipur, India; ^2^Rajiv Gandhi Centre for Diabetes and Endocrinology, J.N Medical College, Aligarh Muslim University, Aligarh, India; ^3^School of Public Health, Post Graduate Institute of Medical Education and Research, Chandigarh, India

**Keywords:** diabetes management, 3E model, cost-effectiveness, machine learning, patient empowerment, natural language processing, long-term projections, medication patterns

## Abstract

**Background:**

This study investigated the cost-effectiveness and clinical impact of the 3E model (education, empowerment, and economy) in diabetes management using advanced machine learning techniques.

**Methods:**

We conducted an observational longitudinal descriptive analysis involving 320 patients, who were grouped into intervention and control groups over a 24-month period.

**Results:**

The 3E model demonstrated significant cost reductions, with the intervention group achieving a 74.3% decrease in total costs compared to 41.8% in the control group while maintaining the same level of glycemic control. Machine learning models, including random forest and K-means clustering, were used to identify key factors influencing treatment costs and to segment patient subgroups that were most responsive to the intervention. Natural language processing techniques revealed medication patterns associated with greater cost reductions. Long-term projections using ensemble methods (such as XG Boost, Exponential Smoothing, and Prophet) predicted that, on average, each year contributes approximately 20% to the total cumulative savings over 5 years. No significant correlations were observed between cost reduction and socioeconomic factors, gender, or age, suggesting the broad applicability of the 3E model.

**Conclusion:**

These findings demonstrate the potential of the 3E model to achieve significant reductions in diabetes management costs without compromising care quality, highlighting its value for healthcare policy and resource allocation in chronic disease management.

## Introduction

Diabetes mellitus is a well-known non-communicable disease that poses a significant threat to the global community. It is characterized by high blood glucose levels that lead to various health challenges and represents a global health crisis. The International Diabetes Federation (IDF) estimated that in 2021, 537 million adults were living with diabetes mellitus, and this number is projected to rise to 853 million by the year 2050 (1). This staggering figure not only threatens global healthcare systems but also destabilizes economies due to the immense financial burden of diabetes care. Diabetes mellitus has a multifaceted economic impact, encompassing both direct and indirect medical costs (2). These costs arise from reduced productivity, lost income, and diminished quality of life (QoL) (3). A 2017 study by Bommer et al. estimated that diabetes mellitus imposed a global burden of $1.3 trillion, which was equivalent to 1.8% of the gross domestic product (GDP) in 2015 (4). The authors also predicted that this figure would increase to $2.1 trillion by 2030 (4). These facts highlight the urgent need for effective cost management strategies.

Conventional strategies for managing diabetes mellitus have primarily relied on both pharmacological and non-pharmacological interventions (5). While these approaches effectively control glycemic levels, they impose a significant economic burden on patients due to the high costs of medications and routine clinical care (5). Furthermore, the American Diabetes Association (ADA) standards of medical care (6) emphasize patient-centered care and self-management education as cost-effective alternatives that can be implemented globally.

With the advent of recent technologies and data-driven approaches in the management of diabetes mellitus, it is now possible to detect the trajectory of the disease. Artificial intelligence (AI) and machine learning (ML) have emerged as powerful tools for forecasting outcomes, delivering personalized treatment plans, and optimizing resources in low-cost settings (7–11). Once implemented, these innovations can enhance efficiency while reducing healthcare expenditures. Contreras et al. predicted micro- and macro-vascular complications with blood glucose levels with a machine learning approach (12). However, this highly sophisticated technology is not integrated with patient management and needs to be explored in detail.

In this context, the present study introduces the 3E model—education, empowerment, and economy—a novel approach in the management of diabetes mellitus using advanced data analytics to improve patient-oriented outcomes (13). This 3E model emphasizes the impact of education and empowerment on the economy of patient care, promoting cost-effective practices to improve patient outcomes and reduce the burden on healthcare systems. The 3E model helped achieve glycemic control and reduced direct and indirect costs while it simultaneously leveraged ML algorithms to optimize treatment strategies and resource allocation (14).

This study aims to address existing gaps by integrating advanced machine learning strategies with patient-centered outcomes in the assessment of clinical and economic impact using the 3E model.

## Methodology

### Study design

The present observational longitudinal study, based on education and empowerment and the development of the 3E model, was conducted in India. The inclusion criteria for the enrollment of study participants include the following: (i) adults aged 25–65 years, regardless of sex; (ii) diagnosis of type 2 diabetes mellitus within the past 6 months; (iii) fasting plasma glucose (FPG) ≥ 126 mg/dL (7.0 mmol/L; no caloric intake for at least 8 h) OR 2-h plasma glucose ≥ 200 mg/dL (11.1 mmol/L) during a 75-g oral glucose tolerance test (OGTT) OR HbA1c ≥ 6.5% (48 mmol/mol), measured using a standardized assay (NGSP-certified and traceable to DCCT) OR random plasma glucose ≥ 200 mg/dL (11.1 mmol/L) in individuals with classic symptoms of hyperglycemia or hyperglycemic crisis; (iv) not requiring insulin therapy at diagnosis (to distinguish from latent autoimmune diabetes in adults or severe T2DM); and (v) ability to provide informed consent and comply with study procedures. Study participants were excluded if they met any of the following criteria: (i) diagnosis of type 1 diabetes mellitus, gestational diabetes, or T2DM diagnosis, which occurred more than 6 months before the study; (ii) undergoing insulin therapy; (iii) eGFR <30 mL/min/1.73 m^2^; (iv) active malignancy or undergoing chemotherapy/radiation; (v) psychiatric illness; (vi) pregnant or breastfeeding women; (vii) alcohol or drug dependence; (viii) presence of micro-vascular complications; and (ix) patients who failed to attend one or more of the three scheduled study sessions.

### 3E implementation strategy

The enrolled participants were given education (one session per week with a duration of 2 h for each session) about the awareness of diabetes, personal counseling, general knowledge about diabetes, coexistence and emotional problems associated with the disease, potential complications, self-monitoring of glucose, motivation, food preparation skills, physical exercise, and quality of life. Additionally, these sessions emphasized the importance of social support, all guided by diabetic educators. The educational model includes printed booklets in the participants’ local languages, multimedia animations, audiovisual presentations, group discussions, one-on-one education, short films, quizzes, experiential learning, carbohydrate identification exercises, and workshops.

Empowerment sessions were conducted by diabetic educators for the enrolled participants. Emphasis was placed on each individual’s cognitive, biophysical, psychological, and social aspects, assuming that a person’s values, beliefs, and opinions must be respected and considered. Emphasis was given to personal strengths rather than the deficits of the patient. Diabetic educators established goals for glycemic control with the participants, but that was slightly negotiable depending on the behavior and mutual agreement. The participants were focused on the development of a responsible attitude for attending regular sessions with diabetic educators. Educators facilitate this process by helping patients explore problems, express feelings, develop alternative options, consider the consequences of various options, and make appropriate decisions. Long-term motivation for being healthy (maintaining strict glycemic control) was provided to participants. The detailed implementation strategy is mentioned in Supplementary File 1.

### Post-implementation data collection

A structured questionnaire was administered to collect baseline and endline data on complete economic profiles, sociodemographic details, history of diabetes, family history of diabetes, other disease-related profiles, adherence to prescribed management questions, and reasons for non-adherence. HbA1c was measured every 3 months after every enrolled subject’s post-education and empowerment sessions. A regular 3E model was implemented until the desired population’s glycemic control was achieved. The assessment of baseline and endline costs for diabetes care before and after the implementation of the education intervention was conducted to determine the cost-effectiveness. This analysis evaluated the costs (direct and indirect) and health effects of specific interventions.

### Cost and clinical outcome analysis

To assess the 3E model’s impact on diabetes management, we conducted a 24-month longitudinal study comparing 160 patients receiving the intervention and 160 controls receiving standard care. Data were collected at baseline, 3, 6, 18, and 24 months, with primary outcomes including total treatment costs (which comprised drug expenses, laboratory fees, equipment costs, and travel/parking expenses) and clinical efficacy (HbA1c levels). Each cost component was analyzed separately to evaluate economic effects, while glycemic trends were monitored to assess therapeutic effectiveness.

We computed the mean and standard deviation of total costs, drug costs, and laboratory test costs for each group at each time point. To determine the statistical significance of cost differences between the groups, we performed independent samples t-tests at each time point. Effect sizes were calculated using Cohen’s d. We also calculated the percentage reduction in costs from baseline to 24 months for both groups. HbA1c levels were compared between the two groups using an independent samples t-test to assess any differences in glycemic control.

We performed Pearson’s correlation analysis to investigate the association between cost reduction and HbA1c levels. All statistical analysis was conducted at a significance of *p* < 0.05 with Python 3.8 with the SciPy library (version 1.6.0). Data manipulation was performed through Pandas (version 1.2.0), and visualization was conducted with Matplotlib (version 3.3.3).

### Socioeconomic and age-related changes with respect to gender

To examine the effect of socioeconomic factors, age, and gender on treatment outcomes, we performed Pearson’s correlation analysis. The correlation between monthly income and cost reduction between the study groups was also analyzed using the coefficient. Furthermore, gender-based analysis was performed to compare the cost reduction using independent samples t-tests.

### Cost-effectiveness analysis

We performed cost-minimization analysis to evaluate the cost-effectiveness of the intervention. We compared the average total cost (drugs and laboratory costs) at baseline and 24 months after the interventions were provided. The cost was compared between the intervention and control groups. Furthermore, incremental cost savings were estimated by calculating the difference in cost savings between the two groups.

To calculate the costs associated with the 3E model implementation, we assumed a cost of 1,000 INR per patient (assuming the cost of educational materials and necessary resources for implementing the 3E model). We later estimated the intervention’s net cost savings by subtracting the implementation cost from the incremental cost savings.

### Long-term saving projection

We used the integration of advanced machine learning and statistical models and the XG Boost tool for 2 years to collect data. This gradient-boosting algorithm is capable of capturing complex, non-linear associations and smoothing data exponentially. Furthermore, mean squared error (MSE) and R-squared (R2) were used to evaluate each model’s performance and gauge its fit to the collected data. We later developed an ensemble prediction by averaging the outcomes of all three models and then further utilized it to calculate the projected cost for both study groups over a 5-year period. This allowed us to predict the long-term impact of the 3E model.

### Feature importance analysis and patient stratification

We examined datasets of 320 diabetes mellitus patients distributed among two groups. We analyzed the major contributory factors affecting the treatment cost using a Random Forest Regressor model (15). We performed equal weighting, and then K-means clustering was used to segment patients based on their treatment outcomes and characteristics (16). Pearson’s correlation was performed to identify the association between study variables.

### Medication pattern analysis using NLP

The natural language processing (NLP) method was used to investigate the association between medication patterns and cost components between study groups (17). The NLP methodology we adopted includes data preprocessing to clean the data for special numbers and characters and convert the text to lowercase. We applied the ‘TREATMENT RECEIVED DRUGS’ column for preprocessing. Our analysis used Count Vectorizer to convert preprocessed datasets into a document term matrix, limiting them to the top 100 features. Subsequently, we applied latent Dirichlet allocation (LDA) with five topics to screen common medication trends and assigned each patient an LDA score (18). All analyses during the study modeling were performed using Python 3.10, employing libraries including Pandas for manipulation, sci-kit-learn and xg boost (ML models), Matplotlib for visualization, and Stats models for time series analysis.

## Results

### Cost and clinical outcome analysis

**Figure 1:**
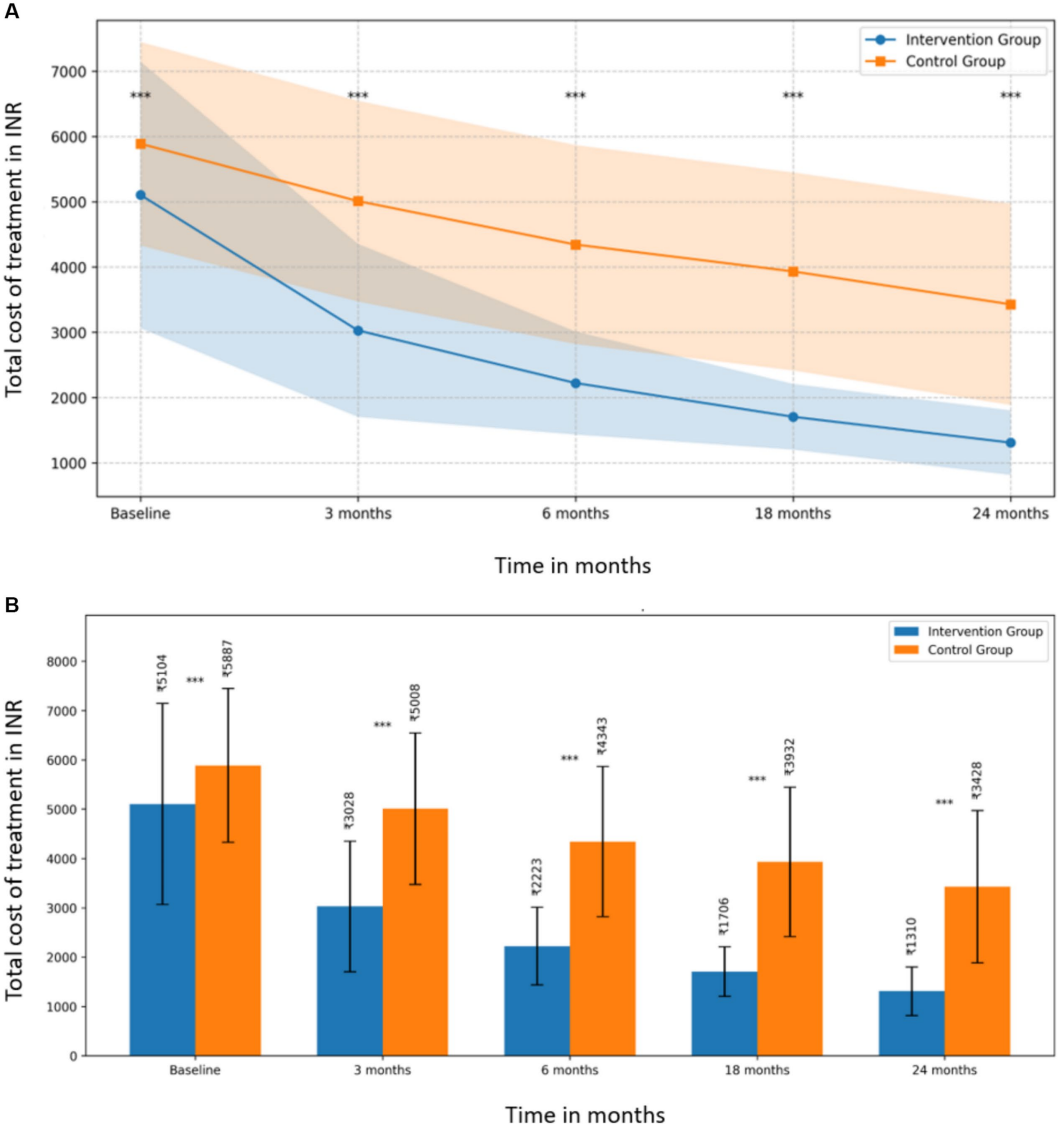
**(A)** Total treatment cost comparison between intervention and control groups over 24 months. **(B)** Mean total costs with standard deviations for intervention and control groups at each time point.

The cost comparison analysis revealed a consistent trend of lower total treatment costs in the intervention group compared to the control group across all time points, with the difference becoming more pronounced over time (Figure 1a).

At baseline, the mean total cost for the intervention group was INR 5,103.63 (SD = INR 2,041.22), while the control group’s mean was INR 5,887.23 (SD = INR 1,557.54), showing a significant difference (t (318) = −3.860, *p* = 0.0001, d = −0.432). This initial difference suggests that the groups were not equivalent at the start of the study, which should be considered when interpreting the results.

The difference between the groups became more substantial at subsequent time points (Figure 1b):

- At 3 months: intervention group (M = INR 3,027.96, SD = INR 1,324.35) vs. control group (M = INR 5,008.29, SD = INR 1,536.53); t (318) = −12.349, *p* < 0.0001, d = −1.381
- At 6 months: intervention group (M = INR 2,222.53, SD = INR 788.62) vs. control group (M = INR 4,343.09, SD = INR 1,523.15); t (318) = −15.639, *p* < 0.0001, d = −1.748
- At 18 months: intervention group (M = INR 1,705.79, SD = INR 502.99) vs. control group (M = INR 3,931.60, SD = INR 1,515.70); t (318) = −17.630, *p* < 0.0001, d = −1.971
- At 24 months: intervention group (M = INR 1,310.24, SD = INR 494.36) vs. control group (M = INR 3,427.80, SD = INR 1,541.76); t (318) = −16.543, *p* < 0.0001, d = −1.850

The effect sizes (Cohen’s d) increased over time, indicating a growing impact of the intervention, with the largest effect observed at 18 months (d = −1.971).

#### Drug cost analysis

**Figure 2:**
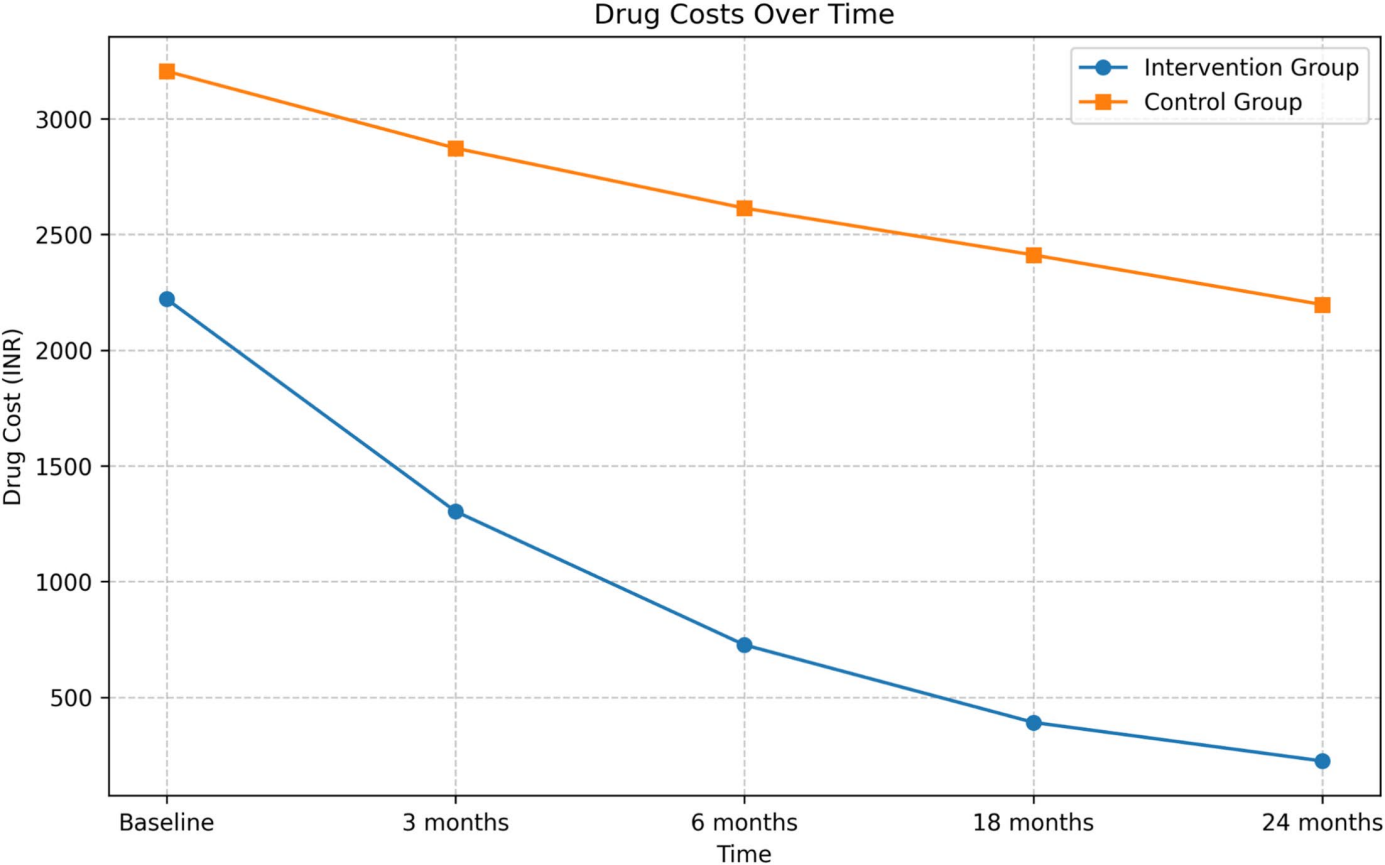
Drug cost reduction in intervention and control groups over a 24-month period.

The intervention group demonstrated a substantial reduction in drug costs over the 24-month period (Figure 2). The mean drug cost for the intervention group decreased from INR 2,220.79 at baseline to INR 224.21 at 24 months, representing an 89.90% reduction. In contrast, the control group’s mean drug cost decreased from INR 3,205.04 to INR 2,196.36, which is a 31.47% reduction.

#### Laboratory test cost analysis

**Figure 3:**
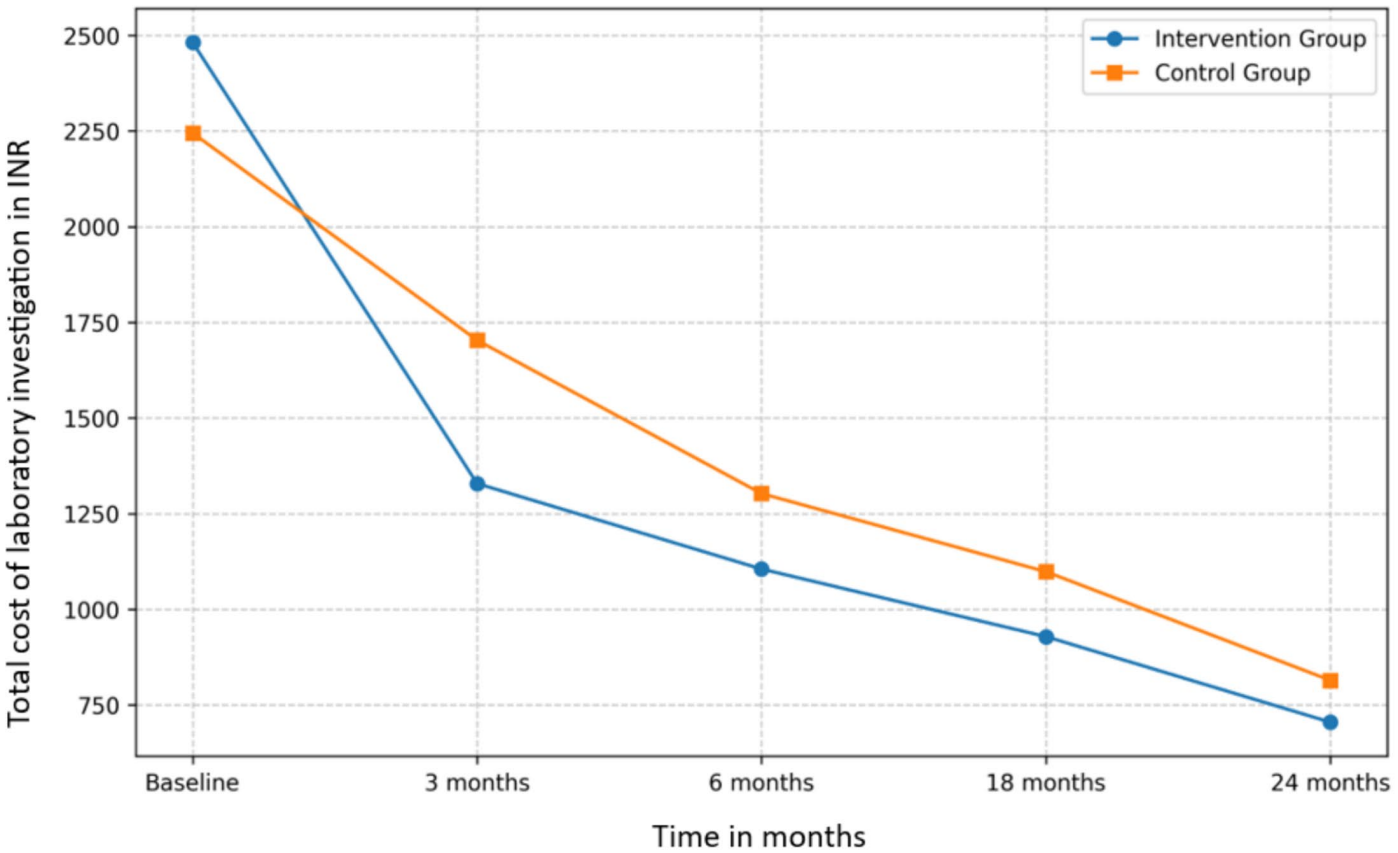
Laboratory test cost reduction in intervention and control groups over a 24-month period.

Both groups exhibited a decrease in laboratory test costs over the study period (Figure 3). The intervention group’s mean laboratory test cost decreased from INR 2,481.25 at baseline to INR 704.88 at 24 months, a 71.59% reduction. The control group’s costs decreased from INR 2,244.54 to INR 814.23, a 63.72% reduction.

#### HbA1c levels analysis

**Figure 4:**
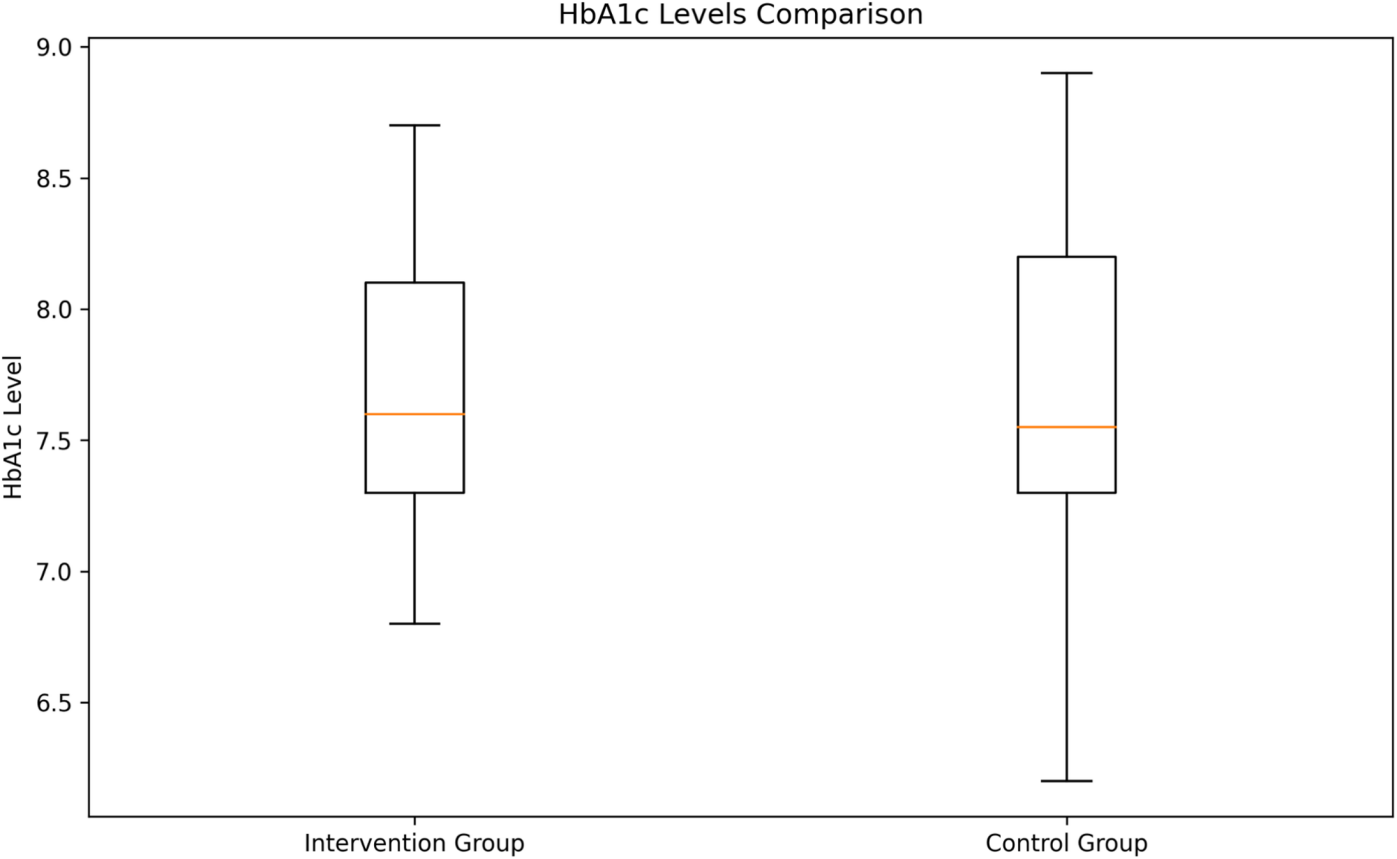
Comparison of HbA1c levels between intervention and control groups.

The analysis of HbA1c levels revealed no significant difference between the two groups (Figure 4). The intervention group had a mean HbA1c level of 7.69% (SD = 0.52), while the control group had a mean of 7.68% (SD = 0.57). An independent samples t-test showed no statistically significant difference between the groups (t = 0.143, *p* = 0.8862).

#### Correlation analysis

**Figure 5:**
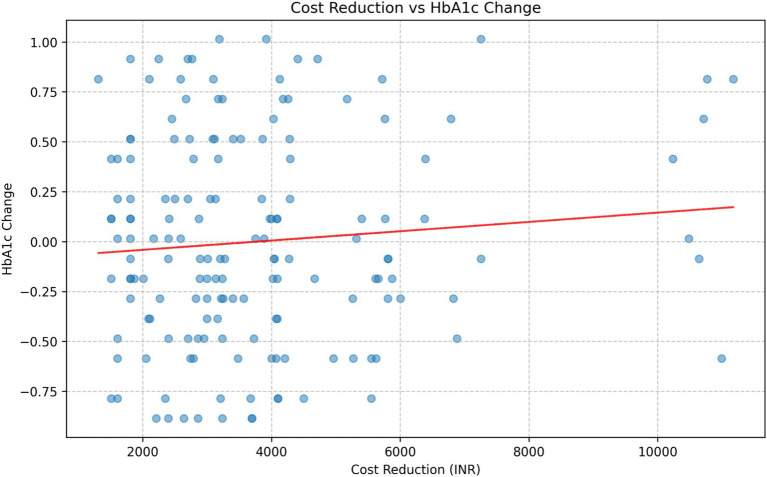
Correlation Between Cost Reduction and HbA1c levels in the Intervention Group.

The correlation analysis between cost reduction and HbA1c change in the intervention group revealed a weak positive correlation (Figure 5). Further analysis of the correlation between total costs and HbA1c levels at each time point showed consistently weak positive correlations: Baseline: r = 0.1005, *p* = 0.2061; 3 months: r = 0.1334, *p* = 0.0925; 6 months: r = 0.1484, *p* = 0.0611; 18 months: r = 0.1328, *p* = 0.0941; 24 months: r = 0.1106, *p* = 0.1638.

None of these correlations reached statistical significance at *p* < 0.05, although the correlations at 3 months, 6 months, and 18 months approached significance (*p* < 0.10).

### Socioeconomic, gender, and age-related analyses

**Figure 6:**
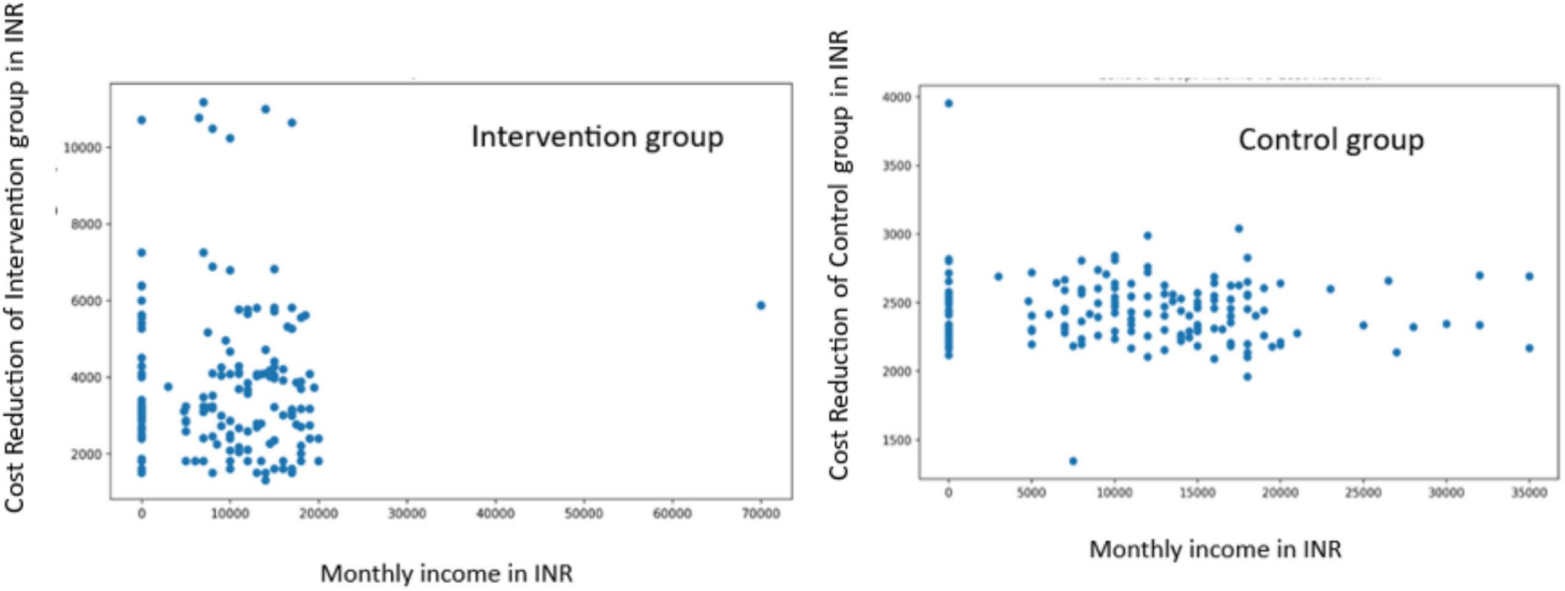
Correlation between monthly income and cost reduction in intervention and control groups.

Socioeconomic Analysis: In the intervention group, we found no significant correlation between monthly income and cost reduction (r = 0.0002, *p* = 0.9980). Similarly, the control group showed a weak negative correlation that was not statistically significant (r = −0.0817, *p* = 0.3044) (Figure 6).

**Figure 7:**
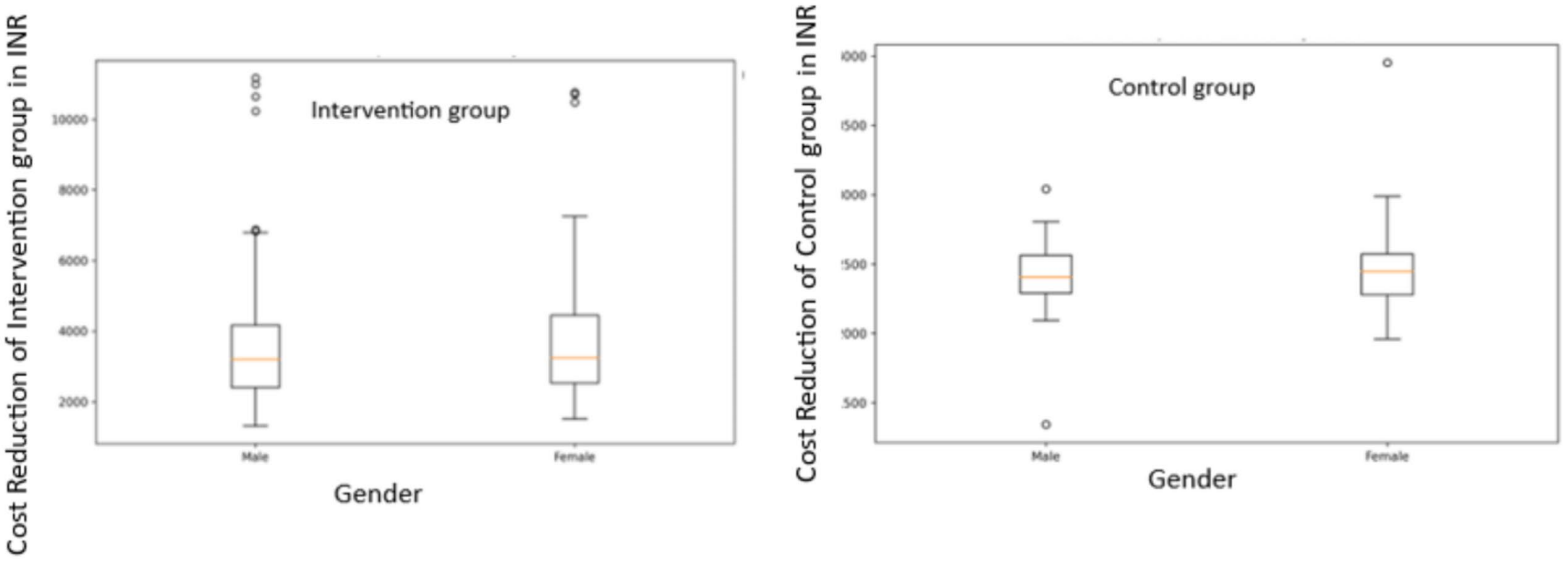
Gender-based analysis of cost reduction in intervention and control groups.

Gender-based Analysis: In the intervention group, male patients showed a mean cost reduction of INR 3706.36 (SD = 1965.53), while female patients had a mean reduction of INR 3,890.05 (SD = 2146.54). The difference was not statistically significant (t = −0.5495, *p* = 0.5834). In the control group, male patients had a mean cost reduction of INR 2410.65 (SD = 228.18) and female patients had a mean reduction of INR 2468.04 (SD = 261.69). Again, this difference was not statistically significant (t = −1.4794, *p* = 0.1410) (Figure 7).

**Figure 8:**
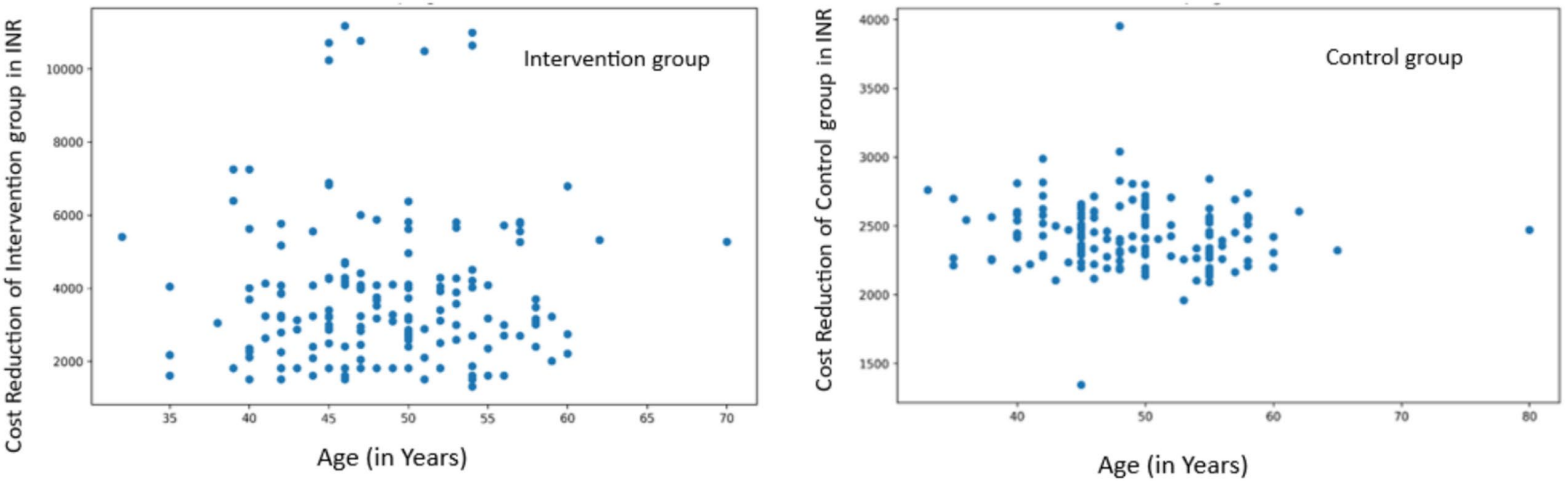
Correlation between age and cost reduction in intervention and control groups.

Age-related Analysis: In the intervention group, we found a very weak positive correlation between age and cost reduction that was not statistically significant (r = 0.0326, *p* = 0.6824). The control group showed a weak negative correlation that was also not statistically significant (r = −0.1042, *p* = 0.1897) (Figure 8).

### Cost-effectiveness analysis

**Figure 9:**
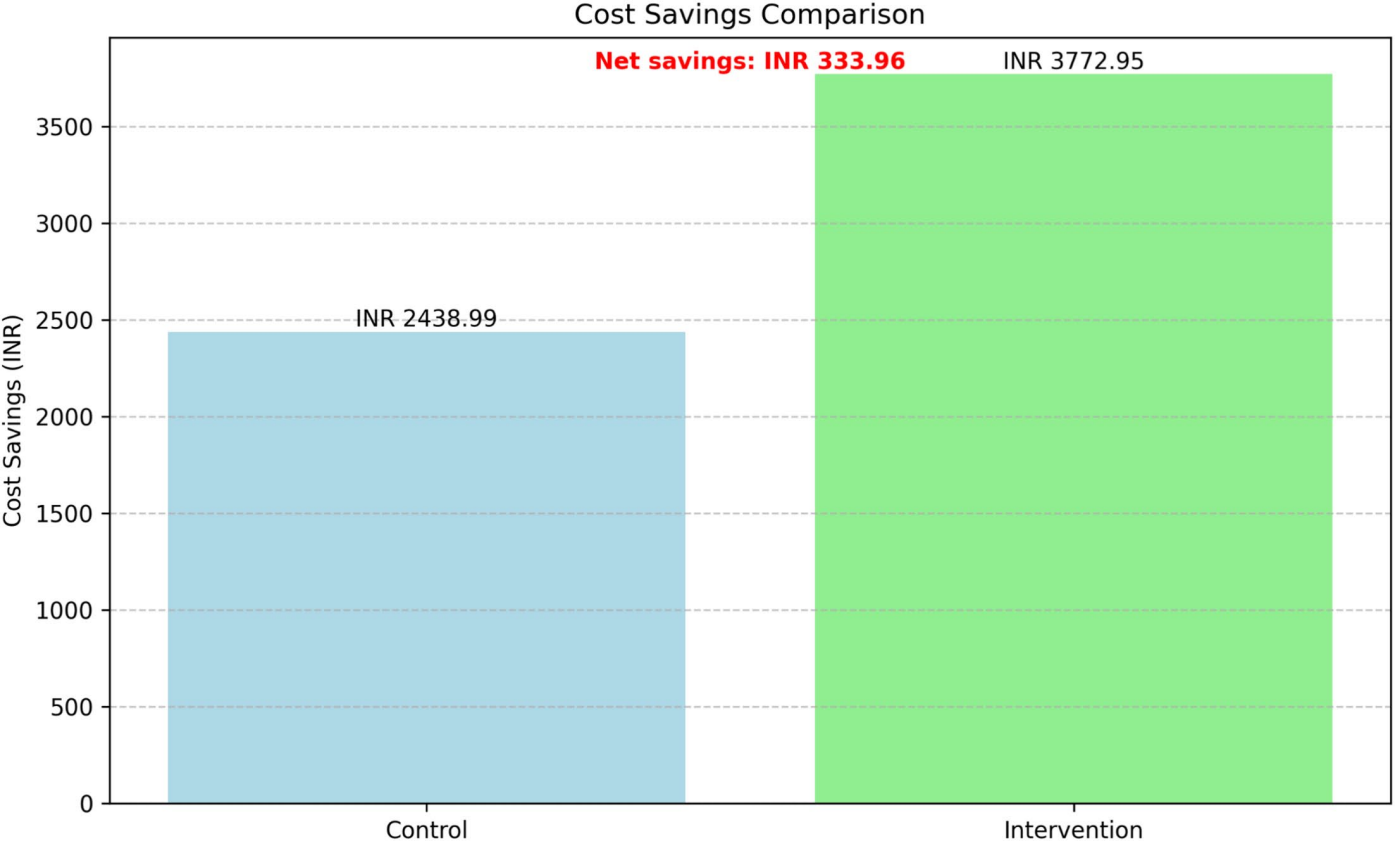
Cost-effective analysis of intervention and control groups.

The cost-effectiveness analysis revealed substantial differences in cost savings between the intervention and control groups while maintaining similar glycemic control (Figure 9). The intervention group demonstrated significantly higher average cost savings (INR 3,772.95) compared to the control group (INR 2,438.99), resulting in incremental cost savings of INR 1,333.96 per patient over the 24-month period.

After accounting for the implementation cost of 1,000 INR per patient, the net cost savings of the intervention were INR 333.96 per patient. The comparison of HbA1c levels confirmed that there was no significant difference in glycemic control between the two groups, as reported in the HbA1c Levels Analysis above.

### Long-term saving projection

The XG Boost model showed a perfect fit for the intervention group with an R2 of 1.00 and an MSE of 0.00. The Exponential Smoothing model also performed well with an R2 of 0.73 and an MSE of 474,633.48. For the control group, the XG Boost model again showed a perfect fit (R2 = 1.00, MSE = 0.00), while the Exponential Smoothing model performed exceptionally well with an R2 of 0.97 and an MSE of 25,263.89.

#### Long-term cost projections

**Figure 10:**
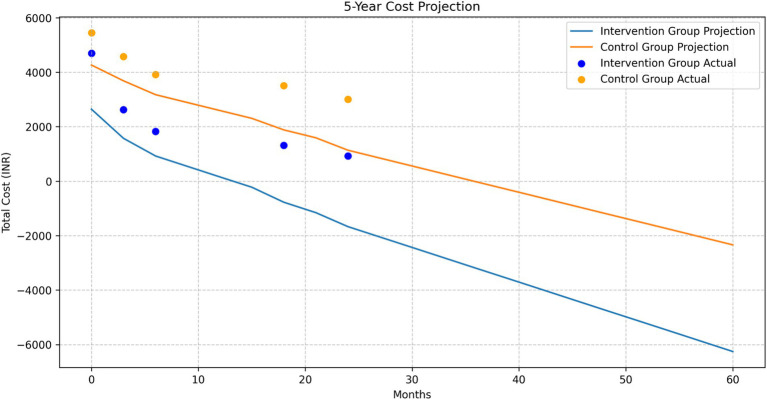
5-year cost projection for intervention and control groups.

The 5-year cost projection showed a continuing trend of lower costs for the intervention group compared to the control group. Both groups showed a decreasing trend in costs over time, with the intervention group maintaining a consistently lower cost trajectory (Figure 10).

#### Cumulative savings

**Figure 11:**
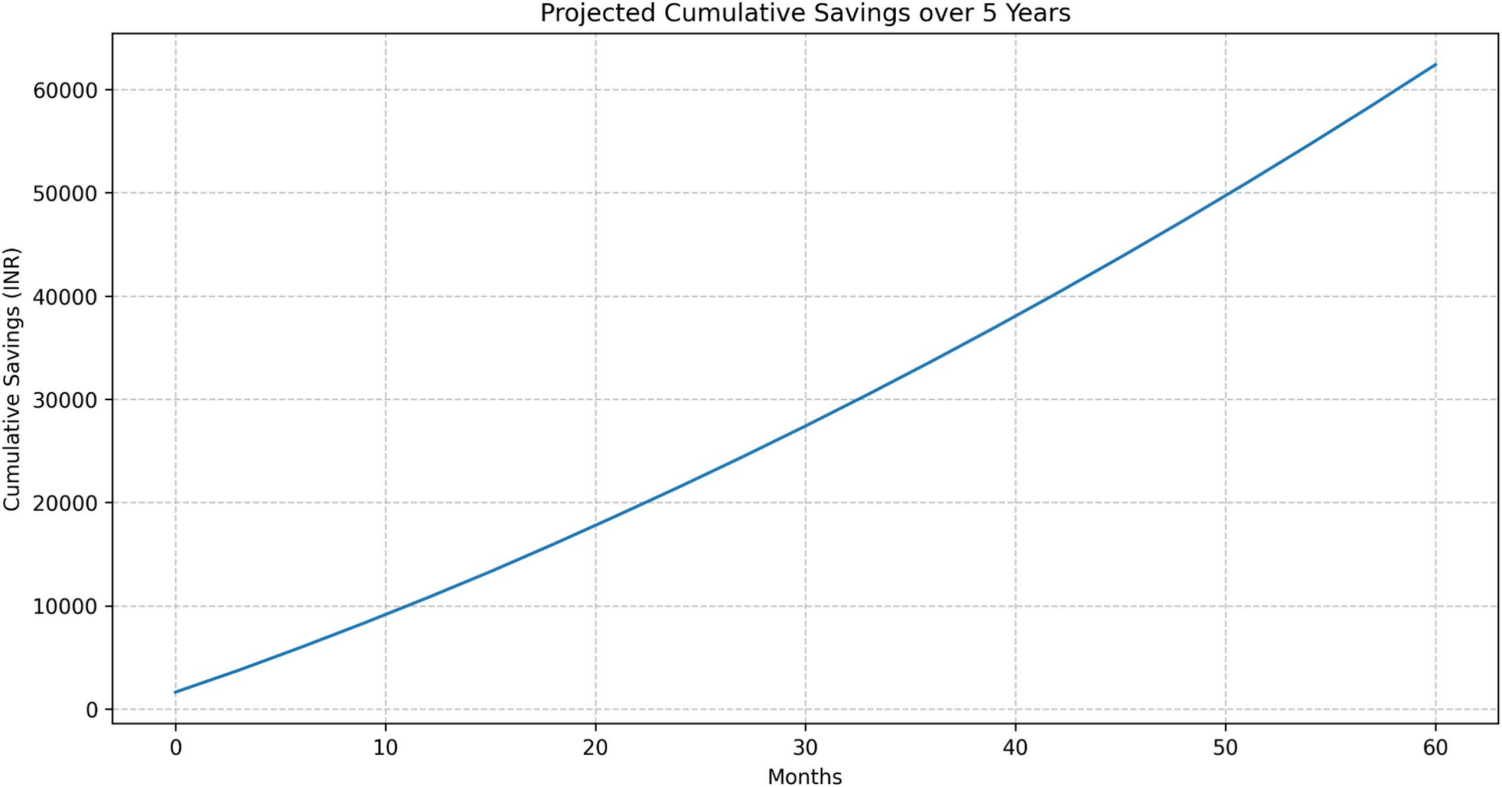
Projected cumulative savings over 5 years.

The projected cumulative savings showed a steadily increasing trend over the 5-year period (Figure 11):

- 1 Year: INR 10,769.11
- 3 Years: INR 33,664.67
- 5 Years: INR 62,409.83

HbA1c Levels: The projected HbA1c levels remained stable and nearly identical between the two groups:

- Intervention Group: 7.69
- Control Group: 7.68

### Feature importance analysis and patient stratification

#### Feature importance

**Figure 12:**
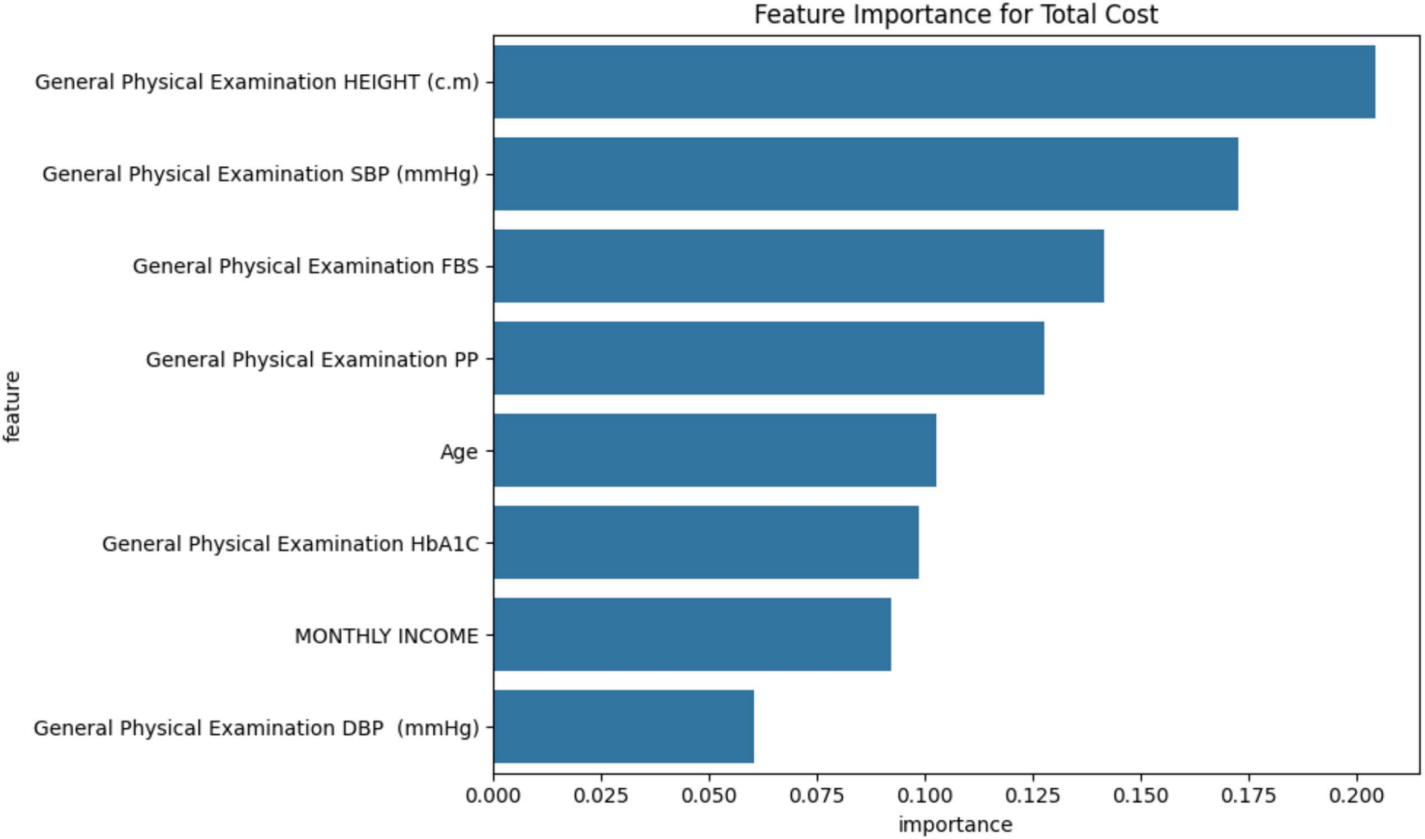
Feature importance analysis: Factors influencing total treatment costs.

The Random Forest model identified the following factors as most influential in determining total treatment costs, in order of importance (Figure 12):

1. Height (20.44% importance)
2. Systolic Blood Pressure (17.28%)
3. Fasting Blood Sugar (14.15%)
4. Postprandial Glucose (12.77%)
5. Age (10.28%)
6. HbA1c (9.85%)
7. Monthly Income (9.21%)
8. Diastolic Blood Pressure (6.03%)

While the Random Forest model identified physiological variables (e.g., blood pressure and glucose levels) as key cost predictors, potential confounding variables such as socioeconomic status, lifestyle behaviors, and healthcare access were also examined. Although these factors showed weak or non-significant correlation in our analysis, their role cannot be overlooked. Lower income may limit treatment adherence, and limited healthcare access can delay interventions, potentially increasing costs. Though not directly measured, lifestyle factors such as diet and physical activity may influence outcomes through metabolic control. Future models should integrate these variables more explicitly to better account for their potentially confounding effects on cost and clinical outcomes.

#### Cluster analysis

**Figure 13:**
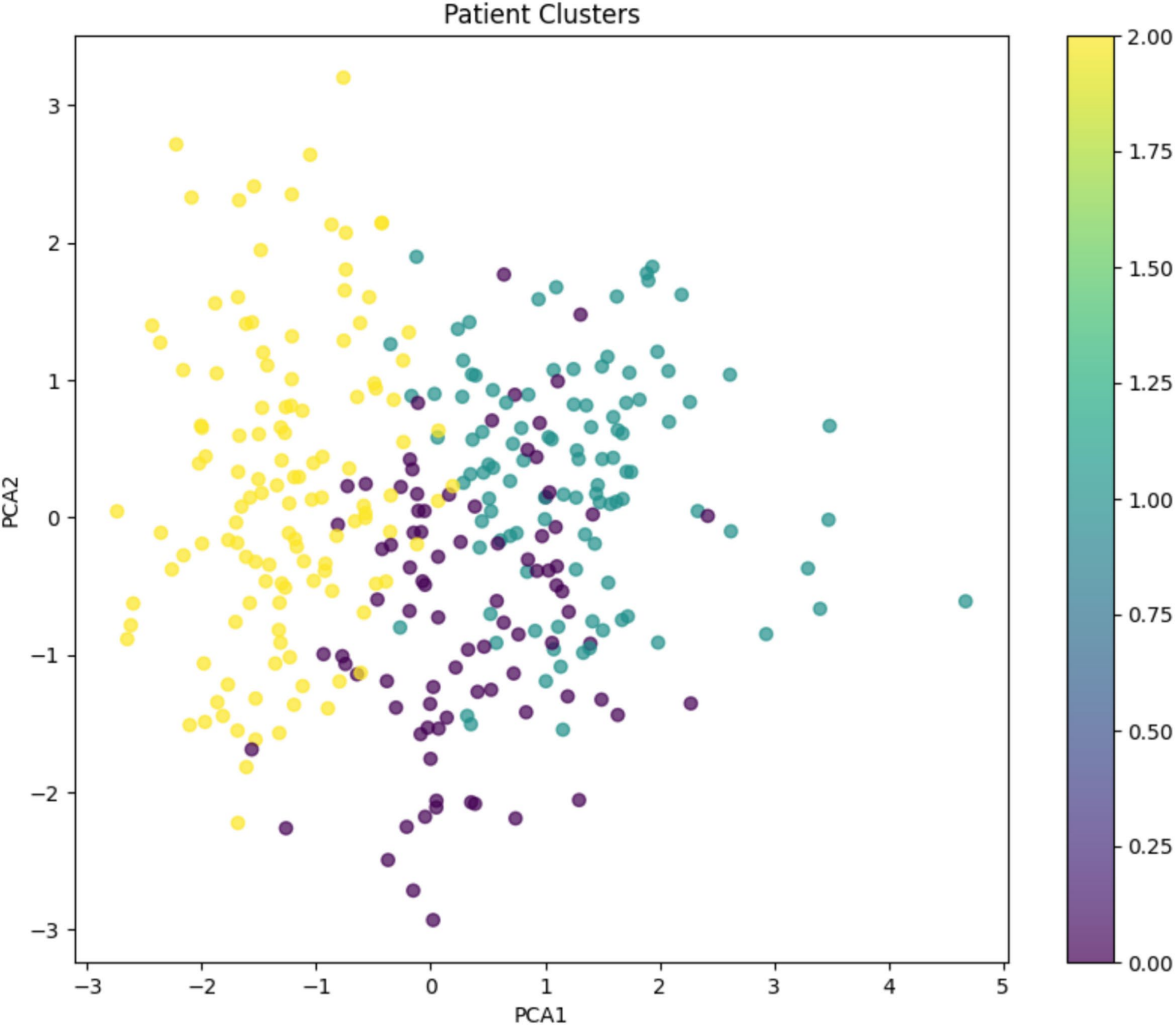
K-means clustering analysis: Patient stratification based on total cost, age, monthly income, and HbA1c levels.

The K-means algorithm identified three distinct patient clusters (Figure 13).

- Cluster 0 (moderate cost and mixed-income): Total average Cost: INR 6,689, age: 48.5 years, monthly income: INR 13,111, HbA1c: 7.69%, predominantly from the intervention group
- Cluster 1 (higher cost and higher income): Total average cost: INR 6,956, age: 52.7 years, monthly income: INR 16,090, HbA1c: 7.71%, predominantly from the intervention group
- Cluster 2 (highest cost and lower income): Total average cost: INR 7,370, age: 45.5 years, monthly income: INR 4,598, HbA1c: 7.65%, predominantly from the control group

#### Correlation analysis

**Figure 14:**
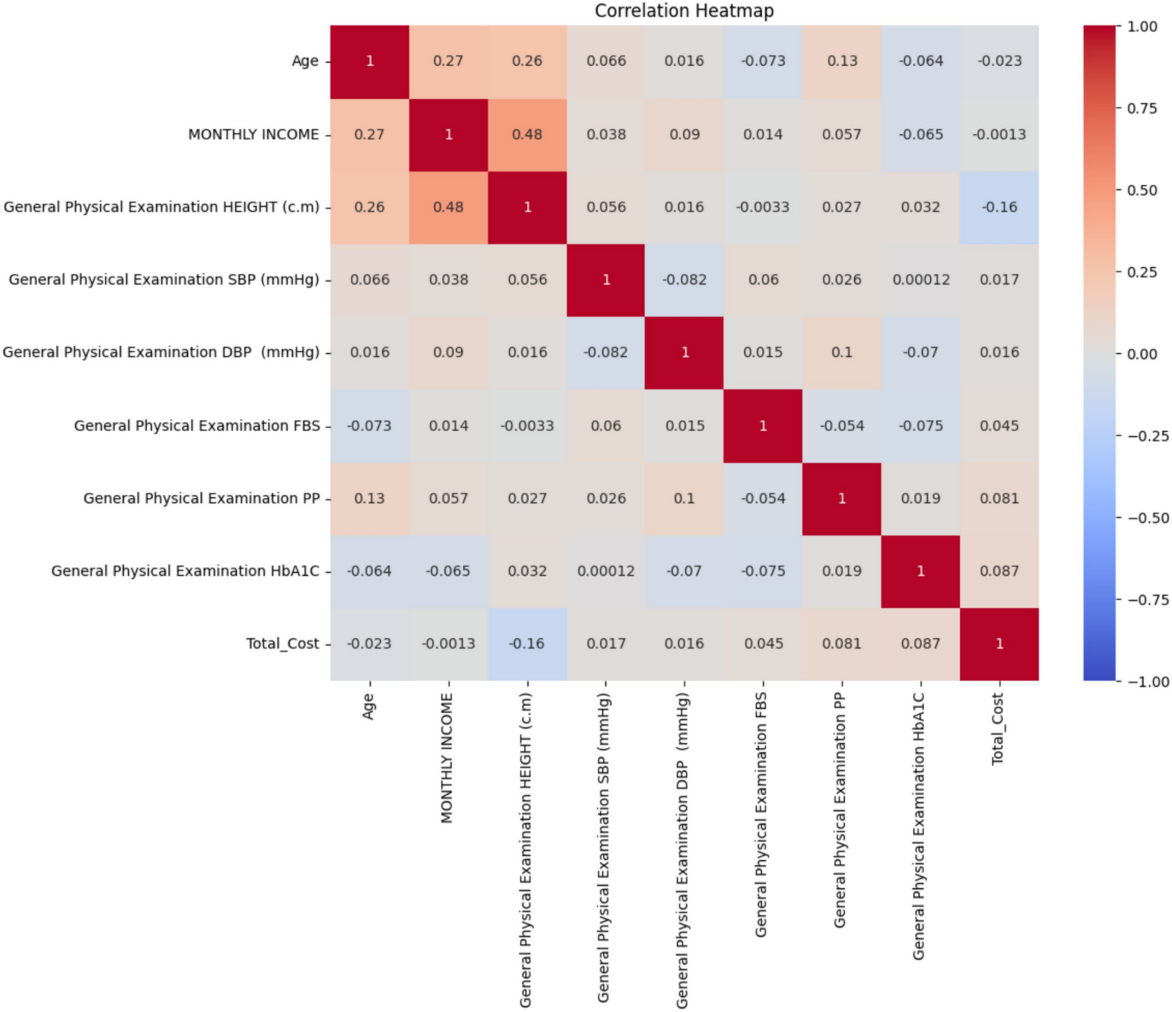
Correlation heatmap of patient characteristics and treatment outcomes.

Key findings from the correlation heatmap include (Figure 14):

1. Age showed a moderate positive correlation with monthly income (r = 0.27) and height (r = 0.26).
2. Monthly income had a moderate positive correlation with height (r = 0.48).
3. Total cost showed very weak correlations with the majority of variables, with the strongest being a weak negative relationship with height (r = −0.16).
4. HbA1c levels showed very weak correlations with all other variables, including total cost (r = 0.087).
5. Fasting blood sugar (FBS) and postprandial glucose (PP) showed weak correlations with other variables, including each other (r = −0.054).
6. Blood pressure measurements (SBP and DBP) showed very weak correlations with other variables and with each other (r = −0.082).

The lack of significant associations between cost savings and gender, income, or age may be attributed to underlying heterogeneity in disease progression, treatment adherence, and healthcare utilization across individuals. Diabetes progression can vary widely regardless of demographic factors, potentially masking cost-related trends. Additionally, the uniform implementation of the 3E model may have reduced disparities by offering consistent education and support across all groups. Variability in individual health-seeking behavior, comorbidity burden, and baseline glycemic control may further diminish the influence of sociodemographic variables. Future stratified analyses may help uncover nuanced patterns and better explain these non-significant associations in larger cohorts. The incremental cost-effectiveness ratio (ICER) was calculated using the formula: ICER = (cost of intervention—cost of control/difference in effect). Based on the total intervention cost of INR 1,310.24 and control cost of INR 3,427.80, the ICER was found to be negative, indicating that the intervention was dominant and cost-effective.

### Medication pattern analysis using NLP

The LDA model identified five distinct medication topics.

- Topic 0: This topic focused on extended-release formulations and combination therapies (e.g., gylcomet and exermet)
- Topic 1: This topic included various medication types, notably some for neuropathy (e.g., gabaneuron)
- Topic 2: This topic emphasized specific diabetes medications and combinations (e.g., dupent and dapanorm)
- Topic 3: This topic centered on common diabetes medications and some cardiovascular drugs (e.g., sitabite and amlong)
- Topic 4: This topic included a mix of diabetes medications and potentially thyroid treatments (e.g., glimfirst and tabthyronorm)

**Figure 15:**
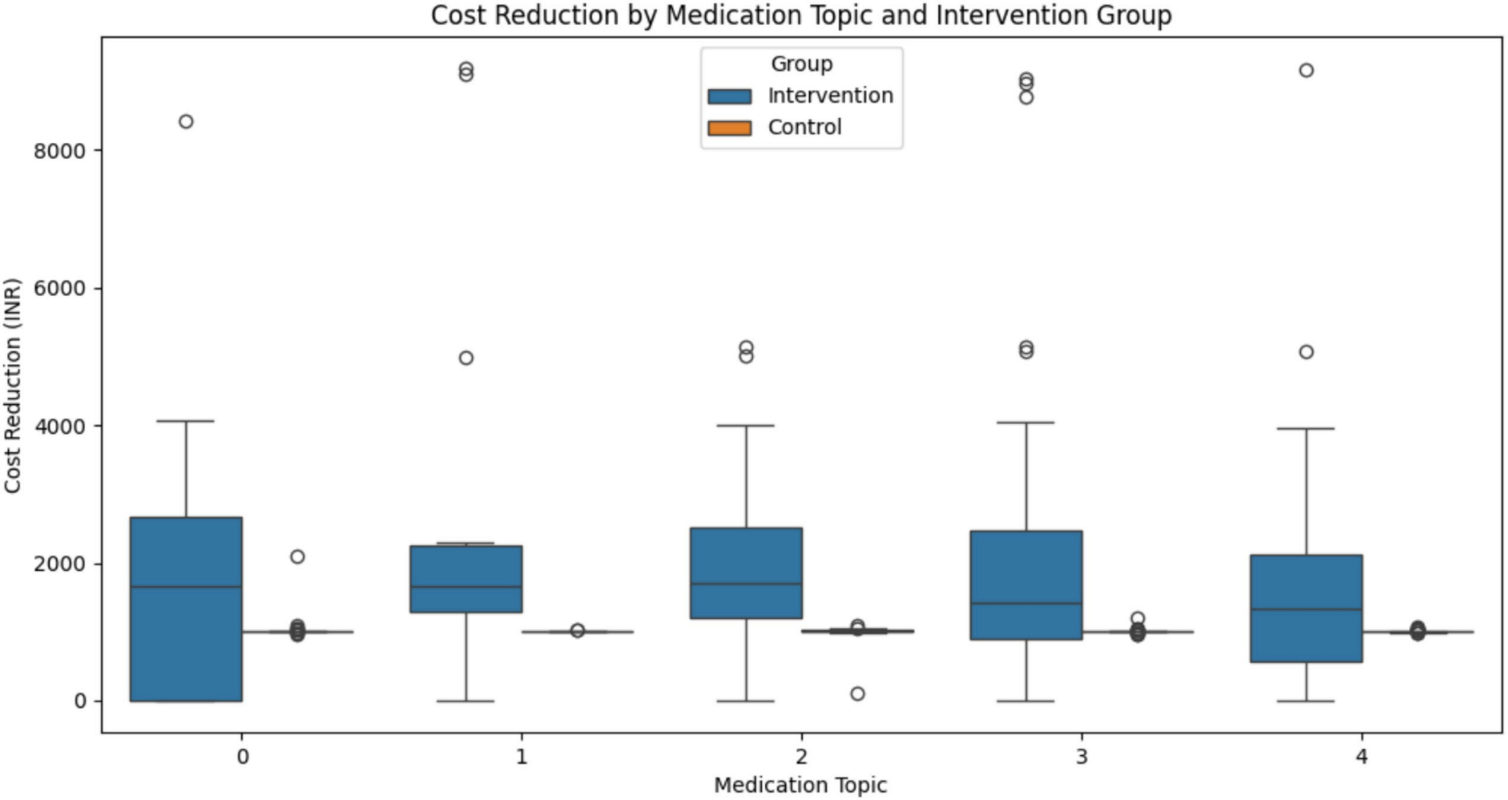
Cost reduction analysis.

Cost Reduction Analysis: All medication topics showed statistically significant differences in cost reduction between the intervention and control groups, except for Topic 4 (Figure 15). The results for each topic were as follows:

- Topic 0: t = 2.759, *p* = 0.0074
- Topic 1: t = 2.351, *p* = 0.0247
- Topic 2: t = 3.840, *p* = 0.0003
- Topic 3: t = 3.384, *p* = 0.0011
- Topic 4: t = 1.880, *p* = 0.0645

Topic 2 shows the most significant difference, suggesting that patients on these medications benefited most from the intervention in terms of cost reduction.

These results provide a comprehensive overview of the impact of the 3E model on treatment costs, clinical outcomes, and medication patterns in diabetes management, highlighting its effectiveness in reducing costs while maintaining glycemic control across various patient subgroups and medication regimens.

## Discussion

The present study reported a significant reduction in cost in the intervention group that received the 3E (education, empowerment, and economy) intervention, along with a notable decrease in HbA1c levels. This study utilized a ML approach to provide a comprehensive understanding of the variables affecting treatment costs and outcomes. Similar observations were made by Chen Y et al., who emphasized the role of empowerment in glycemic control (19). This longitudinal study compared the intervention and control groups over a period of 24 months. The group that received 3E intervention showed a total cost reduction of 74.3% compared to 41.8% in the control group (*p* < 0.05), with the largest effect size observed at 18 months (Cohen’s d = −1.971). The results of this study showed that the 3E model is cost-effective and has a positive impact on patient-oriented outcomes.

Random forest (RF) analysis showed that systolic blood pressure and fasting blood glucose levels are among the major predictors of the total cost in the present study’s intervention. Our results are in complete agreement with those of a previously published study on a Japanese population (20). These findings suggest that physiological contributors are crucial in determining the total treatment costs, as these variables are highly correlated with the comorbidities and disease severity. Furthermore, the K-means clustering analysis showed lower and moderate cost clusters among the patients in the intervention group (21). This concluded that the 3E model may be beneficial for certain patients with moderate monthly income and younger age. The high cost observed among the aged patients in the high-income clusters indicated that age-associated factors may partially offset the cost savings in the intervention group (22).

The K-means clustering analysis revealed three distinct patient groups, with the intervention group dominating the lower and moderate-cost clusters. This suggests that the 3E model may be particularly effective for certain patient profiles, potentially those with moderate income and younger age. The higher costs observed in the older, higher-income cluster (predominantly intervention group) indicate that age-related factors may partially offset the cost-saving effects of the intervention (22–26).

Our NLP analysis of medication patterns uncovered significant variations in cost reduction across different medication regimens. The most substantial cost reductions were observed in patients taking specific diabetes medication combinations (Topic 2), suggesting that the 3E model may be particularly effective in optimizing the use of these drugs, possibly through improved adherence or more efficient dosing.

The long-term projections generated by our ensemble of machine learning models (XG Boost, Exponential Smoothing, and Prophet) predict continued accumulation of savings over a 5-year period, reaching an average of 20% savings per patient. This projection supports the long-term sustainability and cost-effectiveness of the 3E model.

However, the associated limitation of the present study includes overfitting in some models because of limited available time points and the assumptions of HbA1c levels during long-term projections. However, the baseline cost characteristics between the non-intervention and intervention groups suggest potential selection bias.

The findings of the present study align with the observations made by the authors of the previously published study, in which they reported a positive impact of education and empowerment on cost and HbA1c management (13). However, our study extends further to quantify long-term cost benefits by leveraging ML algorithms and integrating them with data-driven approaches.

## Conclusion

The present study concluded that the 3E (education, empowerment, and economy) intervention was significantly cost-effective for managing both direct and indirect costs associated with diabetes mellitus. The intervention was also effective in the significant reduction of HbA1c levels. The 3E model demonstrated a significant reduction of 74.3% in total costs, along-with projected cumulative savings averaging 20% per patient over a 2-year period compared to those who did not receive the intervention. NLP analysis identified medication patterns predictive of cost reduction. These findings highlight the utility of the 3E model across diverse healthcare settings and support the integration of AI- and ML-based data-driven approaches to provide the best patient-centered care and reduce economic burden worldwide, especially in developing countries, while ensuring high standards of care.

## Data Availability

The raw data supporting the conclusions of this article will be made available by the authors without undue reservation.
