# Supplementary material for: Cost-effectiveness of the 3E model in diabetes management: a machine learning approach to assess long-term economic impact
Source: Front Public Health. 2025 May 23;13:1571546. doi: 10.3389/fpubh.2025.1571546 (PMC12141285; doi:10.3389/fpubh.2025.1571546)
Supplement: Supplementary file 2 [file Table_2.docx]

**Supplementary file 1**

| **Week** | **Session** | **Day** | **Topic** | **Mode of Delivery** | **Activities & Tools Used** | **Led By** |
| --- | --- | --- | --- | --- | --- | --- |
| **Week 1** | Session 1 | Day 1 | Understanding T2DM – Causes, Symptoms, Complications | In-person, group teaching | PowerPoint presentation, printed handouts, pre-test knowledge assessment | Diabetes Educator & PI |
|  | Session 2 | Day 4 | Nutrition in Diabetes – Meal planning, Glycemic Index | Demonstration with food models | Interactive meal planning activity, Indian diet charts | Diabetes Educator |
| **Week 2** | Session 3 | Day 8 | Self-Monitoring of Blood Glucose (SMBG) | Hands-on demo | Glucometer use, logbook distribution, instructional video | Diabetes Educator |
|  | Session 4 | Day 11 | Physical Activity in Diabetes | Role-play & group discussion | 10-minute diabetes-safe exercise routine | Diabetes Educator |
| **Week 3** | Session 5 | Day 15 | Managing Hypo/Hyperglycemia | Group quiz & case scenarios | “What to do” cards, symptom recognition quiz | Diabetes Educator |
|  | Session 6 | Day 18 | Medication Management – Adherence & Myths | Demonstration & discussion | Pill box use, adherence calendar creation | Diabetes Educator |
| **Week 4** | Session 7 | Day 22 | Stress, Sleep & Emotional Wellbeing | Relaxation techniques demo | Breathing exercise, emotional sharing circle | Diabetes Educator |
|  | Session 8 | Day 25 | Empowerment & Goal Setting | One-on-one session | SMART goal setting for diet, exercise, glucose targets; Post-test | Diabetes Educator |

Table: Intervention of 3E model implementation plan.
